# Attochemistry Regulation of Charge Migration

**DOI:** 10.1021/acs.jpca.3c00568

**Published:** 2023-02-15

**Authors:** Aderonke S. Folorunso, François Mauger, Kyle A. Hamer, Denawakage D. Jayasinghe, Imam S. Wahyutama, Justin R. Ragains, Robert R. Jones, Louis F. DiMauro, Mette B. Gaarde, Kenneth J. Schafer, Kenneth Lopata

**Affiliations:** ^†^Department of Chemistry, ^‡^Department of Physics and Astronomy, and ^⊥^Center for Computation and Technology, Louisiana State University, Baton Rouge, Louisiana 70803, United States; § Department of Physics, University of Virginia, Charlottesville, Virginia 22904, United States; ∥ Department of Physics, The Ohio State University, Columbus, Ohio 43210, United States

## Abstract

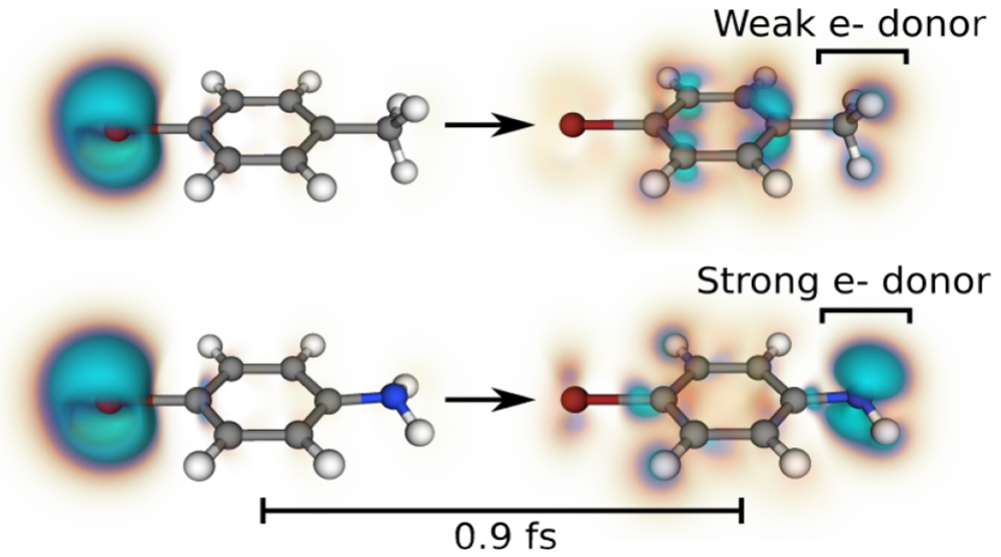

Charge migration (CM) is a coherent attosecond process that involves the movement of localized holes across a molecule. To determine the relationship between a molecule’s structure and the CM dynamics it exhibits, we perform systematic studies of para-functionalized bromobenzene molecules (X–C_6_H_4_–R) using real-time time-dependent density functional theory. We initiate valence-electron dynamics by emulating rapid strong-field ionization leading to a localized hole on the bromine atom. The resulting CM, which takes on the order of 1 fs, occurs via an X localized → C_6_H_4_ delocalized → R localized mechanism. Interestingly, the hole contrast on the acceptor functional group increases with increasing electron-donating strength. This trend is well-described by the Hammett σ value of the group, which is a commonly used metric for quantifying the effect of functionalization on the chemical reactivity of benzene derivatives. These results suggest that simple attochemistry principles and a density-based picture can be used to predict and understand CM.

## Introduction

Charge migration, first identified in the pioneering work of Cederbaum and Zobeley,^1^ is the coherent movement of an electron density hole across a molecule.^2−11^ These holes can be created using neutral excitation^12,13^ or ionization via inner shell^14,15^ or strong field^16,17^ processes. In most cases, coherent charge dynamics involves a superposition of a limited number of states (populated, e.g., via pumping) that beat to give oscillatory motion. This type of dynamics has been simulated previously via methods such as real-time time-dependent density functional theory (RT-TDDFT) for small molecules and chromophores,^18^ complex extended systems,^19^ solvated *para*-nitroaniline,^20^ and silicon carbide,^21^ to name only a few. Coherent processes may be disrupted^22^ or enhanced^23^ by nuclear motion, especially for long-range motion across molecules or at interfaces. In contrast, charge migration (CM) dynamics, as we define it here, is the specific case of a rapidly created localized electron density hole traveling across a molecule in a particle-like way.^1−11^ CM dynamics are postulated to influence longer-time photochemical processes such as photosynthesis, photocatalysis, and light harvesting.^1,13,24^ Furthermore, these dynamics are expected to modulate photochemical reactivity since the distribution of charge in a molecule influences nuclear motion.^25^ Since its discovery, there have been numerous theoretical studies of CM in small molecules using correlated methods^12,15,26−29^ and time-dependent density functional theory,^8,11,30−34^ along with some experimental studies using high harmonic generation and pump–probe ionization methods.^3,4,35^ Organic aromatic molecules are especially promising since they support facile CM due to their conjugated π-electron system,^14,33,36,37^ within which the hole can be viewed as hopping between π-bonds.^8,13^ Although holes may, during a CM event, move with a particular dominant frequency and thus superficially appear similar to a few-state beating, due to the complicated multistate nature of the initial state, CM is better described as a mode in the nonlinear dynamics sense of the word.^31^ As with all coherent dynamics, CM can persist,^14^ be enhanced,^38^ or be disrupted by nuclear motion,^39^ typically on the time scales of a few bond vibrations (sim 10 fs). The emphasis of this work is to determine how chemical functionalization affects the motion of the hole during the first oscillation, before nuclear motion is expected to come into play. Thus, these results are applicable to the specific but highly interesting case of CM in benzene derivatives, which form the foundation of current and future planned CM experiments.

Attosecond coherent electron dynamics in benzene and benzene derivatives has been the focus of much interest.^33,40−42^ In the context of charge migration, Kuleff and co-workers showed that charge migration in benzene survives dephasing for at least 10 fs after the nuclear motion is introduced.^14^ In another, Robb et al. studied CM in paraxylene and showed that the hole swings from one side of the phenyl ring to the other methyl group in 5.2 fs.^43^ Manz et al. studied the restoration of the symmetry of the electronic structure of benzene after being broken, which leads to a periodic charge migration with period *T*.^44^ In contrast, donor–benzene–acceptor (D–B–A) systems have been studied only in an ad hoc manner. Cederbaum and co-workers, for example, showed that charge migration in 4-methyl phenol occurs via a hopping from the methyl group directly to the hydroxyl group in less than 2 fs.^37^ Many questions remain, however, about how charge migration can be modulated by systematic variation in the acceptor, A, group and how this can be understood mechanistically. To address this, in this article we determine the relationship between the structure and chemistry of molecules and the CM dynamics these molecules can support. Due to the wide variety of D–B–A systems, this will help guide future CM experiments.

Here, we present a systematic first-principles simulation study of CM in functionalized bromobenzene derivatives and use this to develop a set of attochemistry principles that draw on simple chemical ideas to predict and understand CM in this family of molecules. Bromobenzene is a good prototypical CM system, as the Br atom supports the creation of a localized hole either via strong-field^16,17^ or inner-shell ionization.^45−47^ Additionally, benzene can be easily introduced into the gas phase and has CM oscillations that survive more than 10 fs, despite the presence of nuclear dynamics.^14^ Moreover, benzene is highly customizable and can be modified with a range of functional groups at the ortho, meta, and para positions to yield stable compounds, many of which are either commercially available or easily synthesized. These compounds are also the building blocks for more complicated systems, such as biomolecules and polycyclic aromatic hydrocarbons (PAHs). Thus, determining structure/CM relationships in the bromobenzene series helps form a bridge between the chemical properties of a molecule and the attosecond dynamics it supports, which can be generalizable to a wide range of systems. These relationships, in turn, will be useful for guiding the choice of molecules for future CM study as well as for interpreting measurements.

## Methods

To simulate CM, we use real-time time-dependent density functional theory (RT-TDDFT) as implemented^18,48,49^ in NWChem.^50^ For all simulations, we use the hybrid PBE0 functional,^9^ cc-pVDZ for H/C and Stuttgart RLC ECP for Br with time steps of 0.2 au (0.005 fs) and 1000 au (24 fs) to propagate the dynamics. We observed that CM was insensitive to the amount of Hartree–Fock admixture in the functional, whereas a nonhybrid functional resulted in a mostly static hole on the Br atom. (See the SI for details.) For the initial state, we use a sudden approximation for the strong-field ionization (SFI) step by creating a hole on the bromine atom at *t* = 0 using constrained DFT (cDFT).^51^ Knowing that SFI from brominated organic molecules results in a Br localized hole,^8,17^ we use cDFT to minimize the energy with the constraint that the Br atom has a +1 charge. In practice, this ionization “simulant” mixes multiple orbitals to give multielectron, multideterminant-like excitations, akin to the self-consistent field (ΔSCF) method for excited states.^52−54^ In a state picture, this localization process puts the molecule in an intricate superposition of ionic states which results in coherent CM dynamics. This bypasses well-known challenges when using TDDFT with adiabatic exchange-correlation functionals to drive systems far from equilibrium.^55−60^ We previously explored the role of the initial hole localization on CM, which can be understood in a nonlinear dynamics framework.^31^

To interpret the resulting dynamics, we use the hole density, ρ^H^(**r**, *t*), computed by subtracting the neutral ground-state density from the time-dependent cation density: ρ^H^(**r**, *t*) = ρ^0^(**r**) – ρ^+^(**r**, *t*). The hole density is then integrated over directions transverse to the CM axis (long axis of the molecule) for easier visualization and for computation of various metrics. To display a clearer time-dependence map that shows the CM modes, we remove the high-frequency contributions in all time-dependent plots of ρ^H^(**r**, *t*) using filtering via convolution with a sin^2^ temporal window with a 0.8 fs total duration. (See the SI for details.)

For the cases that do result in CM, hole density maps can be used to compute a range of physically relevant metrics. The CM time (*t*_CM_) is the time it takes for the hole to travel from the Br atom to the acceptor group −R. The CM distance and speed can be similarly defined^8^ but are not used in this study. To quantify the degree of hole localization on the −R group, we use the hole contrast Γ, a dimensionless quantity that is expected to be correlated with the sensitivity of experimental probes of the density around −R. First, the hole density is computed by integrating the hole density 1 Å above the plane of the molecule and then integrating the hole number on the acceptor −R. This integration selects the part of the density involved in the CM, which mainly occurs in the π system of the molecule. Γ is then obtained by fitting to an offset oscillation with the same frequency as the CM, ,^8^ where *n*_R_^H^ is the number of holes on −R. The hole contrast on −R is given by ratio Γ = *B*/*A*.

## Results and Discussion

**Figure 1:**
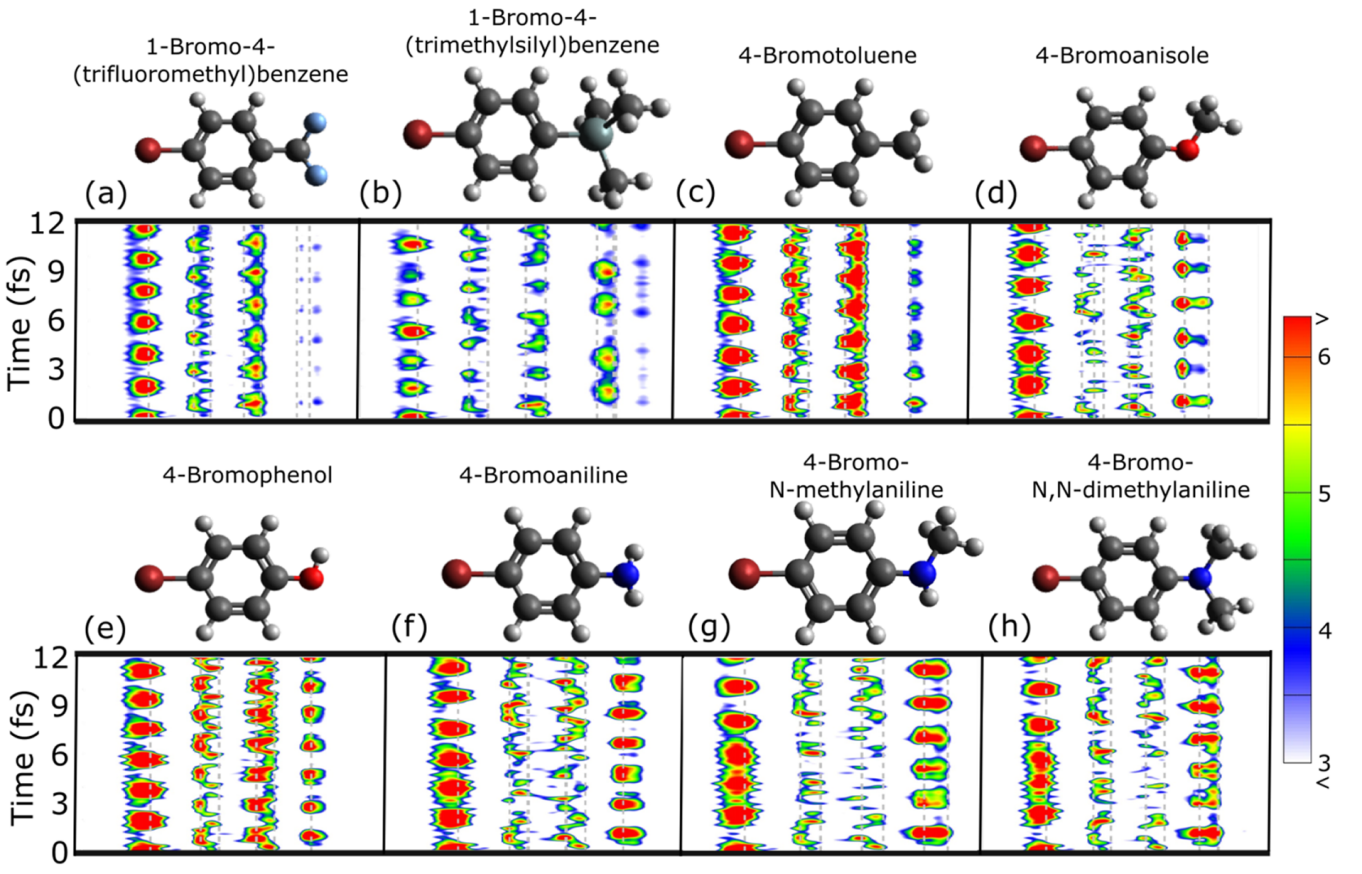
Effect of functional group on CM dynamics in bromobenzene derivatives. Panels (a–h) show the positive part of the time-dependent perpendicularly integrated hole densities following sudden ionization from the Br atom. As the electron-donating strength of the para group increases (a–h), the hole contrast on the end group increases.

Figure 1 shows the integrated hole density time plots for a range of functionalized bromobezene molecules. The functional groups are ordered (a) → (h) by increasing electron-donating strength: 1-bromo-4-(trifluoromethyl)benzene (BrC_6_H_4_CF_3_), 1-bromo-4-(trimethylsilyl)benzene (BrC_6_H_4_Si(CH_3_)_3_), 4-bromotoluene (BrC_6_H_4_CH_3_), 4-bromoanisole (BrC_6_H_4_OCH_3_), 4-bromophenol (BrC_6_H_4_OH), 4-bromoaniline (BrC_6_H_4_NH_2_), 4-bromo-*N*-methylaniline (BrC_6_H_4_NHCH_3_), and 4-bromo-*N*,*N*-dimethylaniline (BrC_6_H_4_N(CH_3_)_2_). In these plots, an oval blob denotes a hole in a particular region around a particular time, i.e., spatiotemporal localization. For example, in Figure 1(e) the hole is mostly on the Br atom at times near 1.86 fs. In contrast, a spatially delocalized hole (at a particular time) is horizontally spread out, such as in Figure 1(f), when the hole is spread across the benzene ring at a time of 0.6 fs. In these systems, a delocalized hole on the ring appears at twice the frequency, which represents the hole crossing the bridging ring twice per period (left to right and right to left). Finally, a vertical/time-axis spread-out hole (not seen in these systems) means the hole is not moving in time. We now qualitatively describe the dynamics in these systems. In Figure 1(a), the −CF_3_ case is distinct in that it does not support CM. This is a consequence of this group’s strong electron-withdrawing strength, which prevents it from accepting a hole. Thus, the dynamics are akin to hole motion from Br into the −C_6_H_4_– ring and back again. As shown in Figure 1(b), the −Si(CH_3_)_3_ (trimethylsilyl; TMS) molecule also has qualitatively different dynamics from the other molecules and involves two distinct modes: a fast (*t*_CM_ = 0.89 fs) Br → phenyl mode and a slow (*t*_CM_ = 1.40 fs) Br → phenyl → TMS mode. This can be understood in terms of the very weak electron-donating strength of TMS, which in the context of CM makes the molecule act as two decoupled regions. Since the calculation of the Hammett parameter involves fitting the hole density above the −TMS group, it is dominated by the slower mode. The remaining molecules all behave similarly and exhibit CM that consists of hole motion from the Br to −R group that takes approximately 1 fs. In these plots, CM appears as a spatially and temporally separated hole on Br, followed by a delocalized hole on the ring, leading to the spatiotemporally localized hole on R.^37^ Since we do not have nuclear motion or other dephasing effects, the CM oscillates indefinitely. Strikingly, as the electron-donating strength of the para-functional group increases, there is a clear increase in the hole density on −R, visible in Figure 1 as increasingly spatiotemporally localized holes.

**Figure 2:**
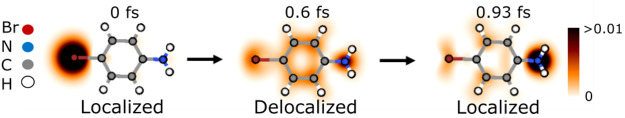
Snapshot of the positive part of the hole density in 4-bromoaniline at 1.0 Å above the plane of the molecule immediately following ionization. The hole undergoes a localized → delocalized → localized charge migration process that takes 0.93 fs.

Before analyzing the relationship between hole contrast and electron-donating strength in detail, we briefly discuss the mechanism by which CM occurs in these systems. Three snapshots of the hole density in 4-bromoaniline are shown in Figure 2. To emphasize the density changes corresponding to CM, as with the contrast calculations, we slice the data at a distance of 1 Å above the plane. The initial localized hole on Br takes approximately 0.6 fs to move into the phenyl ring, at which time it becomes delocalized across the entire ring. The delocalization across the ring (as opposed to π-hopping^8^) is a consequence of the symmetric shape of the molecule, with the phenyl group containing π bonds. After another 0.33 fs, the hole then migrates to the opposite end of the molecule, wherein it becomes localized above/below the NH_2_ group. This overall time scale is consistent with previously reported CM in benzene.^14^ A similar mechanism is observed for all of the molecules that exhibit CM. The observation that the −NH_2_ group supports a local hole at particular times suggests it has a strong hole affinity.

Next, to quantify the hole affinities for various functional groups, we draw a parallel to the conventional chemical definition of electron-withdrawing strength. In substituted benzene rings, each −R group can be assigned a Hammett σ value, which is a way of quantifying how a particular electron-donating or electron-withdrawing group affects the chemical reactivity of a molecule.^61,62^ Hammett σ values have been used historically in physical organic chemistry to draw a relationship between equilibrium or rate constants (*K*) of processes involving one functional group on a benzene ring (e.g., proton dissociation from a carboxylic acid group) and the identity of another functional group in the meta or para position on the ring. The Hammett equation, , has a reaction-specific factor (ρ) that provides insight into the sensitivity of the process to changes in the electron-donating/withdrawing ability and a functional-group-dependent σ factor. More negative σ values correspond to net electron-donating substituents, whereas more positive values correspond to net electron-withdrawing groups. The σ value for hydrogen is typically set to 0 unless necessity dictates otherwise (vide infra). To construct a CM analog of σ, we use a Hammett-like equation, where instead of the chemical reaction rate we use the CM contrast
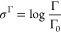
1where σ^Γ^ is the hole contrast σ value, Γ is the hole contrast of a specific R group, and Γ_0_ is the reference hole contrast (−Si(CH)_3_). Typically, the Hammett σ is referenced to benzene (i.e., −R = −H),^61^ but bromobenzene does not support CM and instead involves a hole delocalized across the entire ring. Therefore, we use the hole contrast in 1-bromo-4-(trimethylsilyl)benzene (R = −Si(CH)_3_) for Γ_0_, since trimethylsilyl (TMS) is predicted to be a very weak hole acceptor. Thus, on our scale, σ^Γ^ values that are negative have a better hole affinity than TMS, while functional groups with positive values have a worse hole affinity.

**Figure 3:**
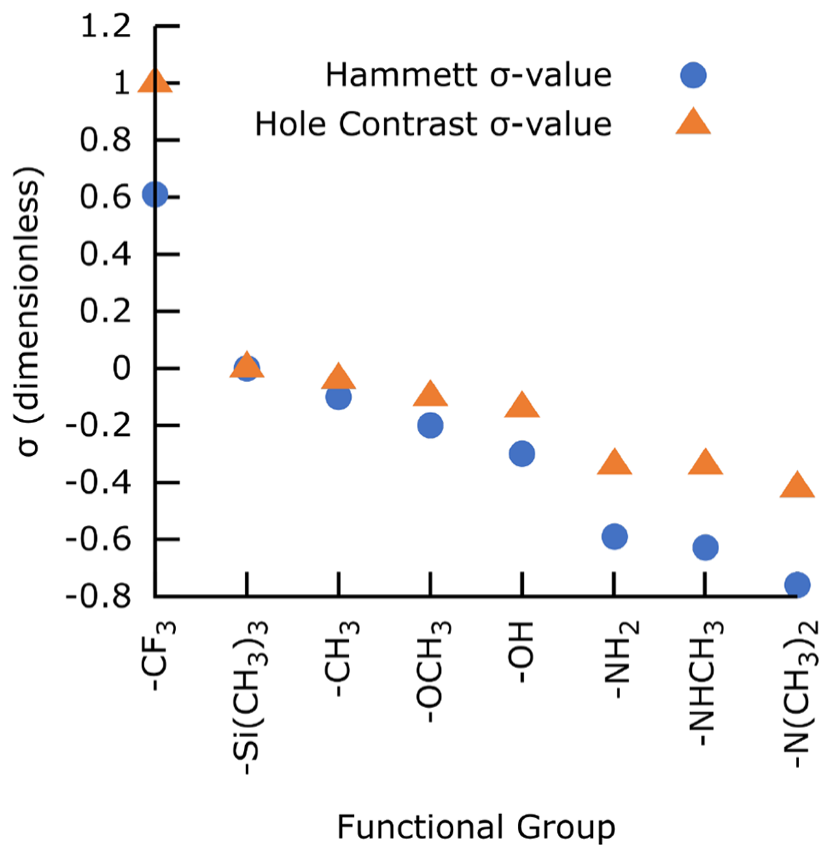
Hammett and hole contrast σ values for *para*-Br–C_6_H_4_–R for various functional groups. More negative values indicate stronger electron-donating strength/hole contrast. The data has been referenced to −Si(CH_3_)_3_ (see the text).

Figure 3 shows the organic chemistry literature Hammett and our computed CM contrast σ values, both referenced to TMS. There is good qualitative agreement between the two quantities, with hole contrast σ^Γ^ decreasing monotonically with decreasing Hammett σ^H^. The Hammett and hole contrast σ values are highly correlated (*R* = 0.998) for the electron-donating groups (i.e., excluding −CF_3_), whereas the CM time and σ values are essentially uncorrelated (*R* = 0.183). See the SI for correlation plots and analysis.

As visible in the dynamics plots in Figure 1, −CF_3_, which has a large positive value (strong electron acceptor) is a bad hole acceptor (high σ^Γ^). −CH_3_, −OCH_3_, and −OH are all relatively weak electron donors (small negative σ^H^) and thus modest CM hole acceptors. This is a consequence of the high electronegativity of these groups being offset by electron donation via resonance. Amine derivatives −NH_2_, −NHCH_3_, and −N(CH_3_)_2_ are highly electron-donating (large negative σ^H^) due to the presence of lone pairs, which they can easily donate, and thus have a correspondingly good CM hole affinity. Furthermore, adding more methyl groups to the N atom increases their electron-donating strength because the −CH_3_ groups donate electron density to the nitrogen, resulting in increased hole affinity for *N*-methylamino and *N*,*N*-dimethylamino relative to amino. This gives a more negative σ value (good hole acceptor), which is visible as dark-red hole densities around the −R group in the dynamics plots in Figure 1(f–h). These results are in agreement with previous studies on differential hole mobilities in doped conjugated molecules, where n- and p-type doping was observed to modulate hole motion.^13^

It is interesting that, at least for the cases presented here, chemical functionalization drastically modifies the hole contrast without significantly affecting the CM time. This makes systematic functionalization a promising avenue for experimental measurements that are sensitive to local electron density at different ends of the molecule (e.g., transient X-ray absorption, high harmonic generation, ionization spectroscopy, etc). On a fundamental level, the surprisingly good correlation between the electron-withdrawing strength and hole contrast is quite illuminating, as it suggests that simple chemical principles that dictate density distributions in molecules can be good predictors of attosecond electron dynamics, at least for CM, which occurs via particle-like motion.

## Conclusions

We have used first-principles simulations to determine the effect of chemical functionalization on halogen-centered strong-field ionization triggered CM in para-functionalized bromobenzene derivatives. In the molecules that do support CM, the observed dynamics involve the movement of the hole across the molecular backbone in a Br localized → ring delocalized → R localized manner, consistent with previous studies that have shown that CM occurs via a hole propagating in the π system of conjugated molecules.^8^ The main observation of this work is that functionalization with groups of varying electron-withdrawing strength only slightly modifies the CM speed but has a pronounced effect on the hole contrast, with strong electron-donating groups supporting higher-contrast CM. Although not studied, we expect similar electron and hole mobilities across the benzene bridge, as reported by Cederbaum and co-workers^13^ who observed that conjugated carbon bridges have similar electron and hole mobilities.

Our findings have numerous implications. From a practical standpoint, they suggest that hole acceptor functional groups can be used as regulators of CM and to enhance the observability of the hole, all without changing the CM time scale. In particular, we predict that the family of bromobenzene derivatives with strong electron-donating functional groups (especially amines) will consist of excellent molecules for experimental measurements that seek to probe the local electron density at different ends of the molecule. On the other hand, from an interpretation standpoint, the simple qualitative relationship between electron-donating strength (Hammet σ value) and hole contrast bolsters the idea that CM can be understood in chemically influenced electron density motion. This density-based attochemistry picture of CM complements emerging resonance-based^8,33^ (hopping of π bonds) and nonlinear multielectron pictures,^31^ both of which describe CM in terms of the electron density alone, without resorting to an ambiguous interpretation in terms of a complicated beating of many states.

## Supporting Information Available

Details regarding the filtering, hole contrast fitting, CM metrics, correlation plots, effects of different DFT functionals, an overview of RT-TDDFT, and the geometries for each molecule (PDF)
