# Supplementary material for: Attochemistry Regulation of Charge Migration
Source: J Phys Chem A. 2023 Feb 15;127(8):1894–900. doi: 10.1021/acs.jpca.3c00568 (PMC9986869; doi:10.1021/acs.jpca.3c00568)
Supplement: Supplementary file 1 — jp3c00568_si_001.pdf [file jp3c00568_si_001.pdf]

Supporting Information for: Attochemistry Regulation of Charge Migration

Aderonke S. Folorunso,[†] François Mauger,[‡] Kyle A. Hamer,[‡] Denawakage D. Jayasinghe,[†] Imam S. Wahyutama,[‡] Justin R. Ragains,[†] Robert R. Jones,[¶] Louis F. DiMauro,[§] Mette B. Gaarde,[‡] Kenneth J. Schafer,[‡] and Kenneth Lopata*,[†,∥]

†*Department of Chemistry, Louisiana State University, Baton Rouge, Louisiana 70803, United States.*

‡*Department of Physics and Astronomy, Louisiana State University, Baton Rouge, Louisiana 70803, United States.*

¶*Department of Physics, University of Virginia, Charlottesville, Virginia 22904, United States.*

§*Department of Physics, The Ohio State University, Columbus, Ohio 43210, United States.*

∥*Center for Computation and Technology, Louisiana State University, Baton Rouge, Louisiana 70803, United States.*

E-mail: klopata@lsu.edu

Filtering

To remove the high-frequency contributions, all time-dependent plots of $\rho^{\mathrm{H}}(r,t)$ were filtered via convolution with a $\sin^2$ temporal window:

$$w(n) = \frac{1}{2} - \frac{1}{2}\cos\left(\frac{2\pi n}{M-1}\right) \qquad 0 \leq n \leq M-1 \tag{1}$$

where M is the number of points in the time signal.

Hole Contrast Fits

Below, we show the time-dependent hole numbers on the $-$R group for each molecule studied, along with the fit used to compute the hole contrast Γ.

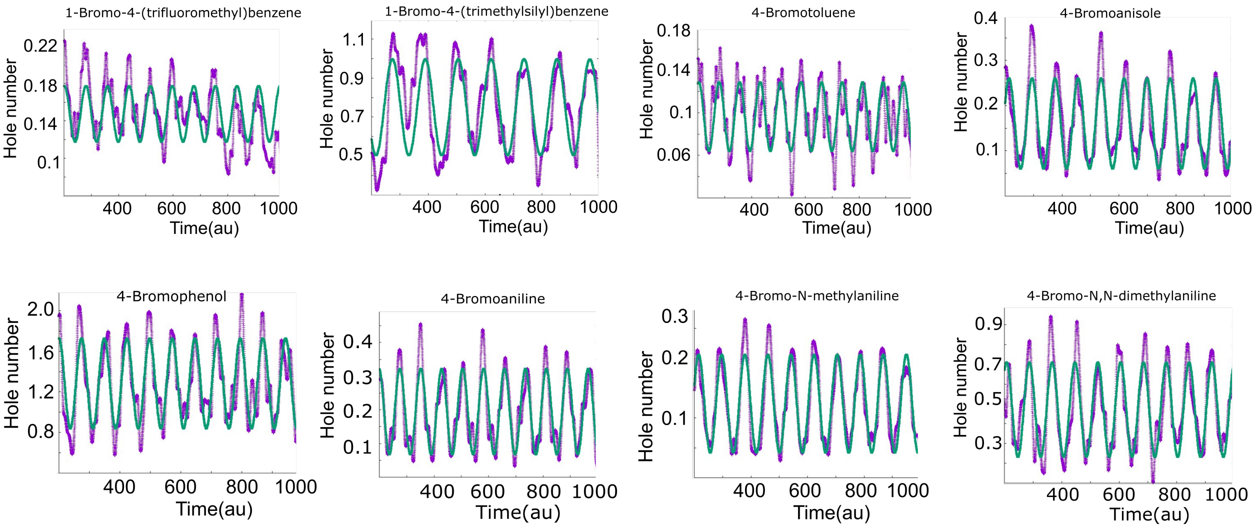

Figure S1: (purple) The hole number $n_{\mathrm{R}}^{H}(t)$ on the $-$R functional group computed from the hole density 1.0 Å above the plane. (green) The fits to a offset oscillation at the charge migration frequency, which are used to compute the hole contrast.

We constructed correlation diagrams to quantify the correlation between our simulated CM metrics and the Hammett sigma values. Fig. S2(a) shows t_{CM} vs Hammett sigma, along with the Pearson correlation coefficient (R). These two quantities are uncorrelated (R=0.183). Fig. S2(b) shows the correlation diagram between the hole contrast sigma and

Table S1: Metrics of CM in bromobenzene derivatives.

**Molecule**	**Functional Group (R)**	$t_{\mathbf{CM}}$	$\sigma^{\mathbf{H}}$	σ^{Γ}
1-Bromo-4-(trifluoromethyl)benzene	$-CF_3$	0.93	0.61	1.00
1-Bromo-4-(trimethylsilyl)benzene*	$-Si(CH_3)_3$	1.40	0.00	0.00
4-Bromotoluene	$-CH_3$	0.93	-0.10	-0.04
4-Bromoanisole	$-OCH_3$	0.98	-0.20	-0.10
4-Bromophenol	$-OH$	0.93	-0.30	-0.14
4-Bromoaniline	$-NH_2$	0.93	-0.59	-0.34
4-Bromo-N-methylaniline	$-NHCH_3$	1.00	-0.63	-0.35
4-Bromo-N,N-dimethylaniline	$-N(CH_3)_2$	0.93	-0.76	-0.42

* The hammett sigma (σ^{H}) and hole contrast sigma (σ^{Γ}) values are calculated using $Si(CH_3)_3$ as the reference (see main text).

the Hammett one. Black dots denote electron-donating groups (negative sigma) whereas the red dot denotes the one electron-withdrawing group ($-CF_3$). We exclude this molecule from the correlation analysis since it does not support CM and behaves qualitatively different from the others in the series. Using only the electron donors (all of which support CM), there is a correlation of R=0.998 between the Hammett and hole values, suggesting an excellent correlation.

Effect of DFT Functional

For the two CM-supporting molecules ($-CH_3$,$-NH_2$), the CM is insensitive to the amount of HF exchange. On the other hand, CM is not observed when using the PBE functional. We attribute this to a poorly localized cDFT hole when using non-hybrid functionals, which are known to overly delocalize charge. Indeed, it has been previously reported that using a hybrid cDFT initial state and a non-hybrid DFT for time propagation results in robust CM.[1] The corresponding sigma contrast values for the 0.50 HF case agree well with the 0.25 case presented in the main manuscript and follow the same trend vs the Hammett sigma value. The charge migration times also agree well, with the 0.5 hybrid cases giving a 9%

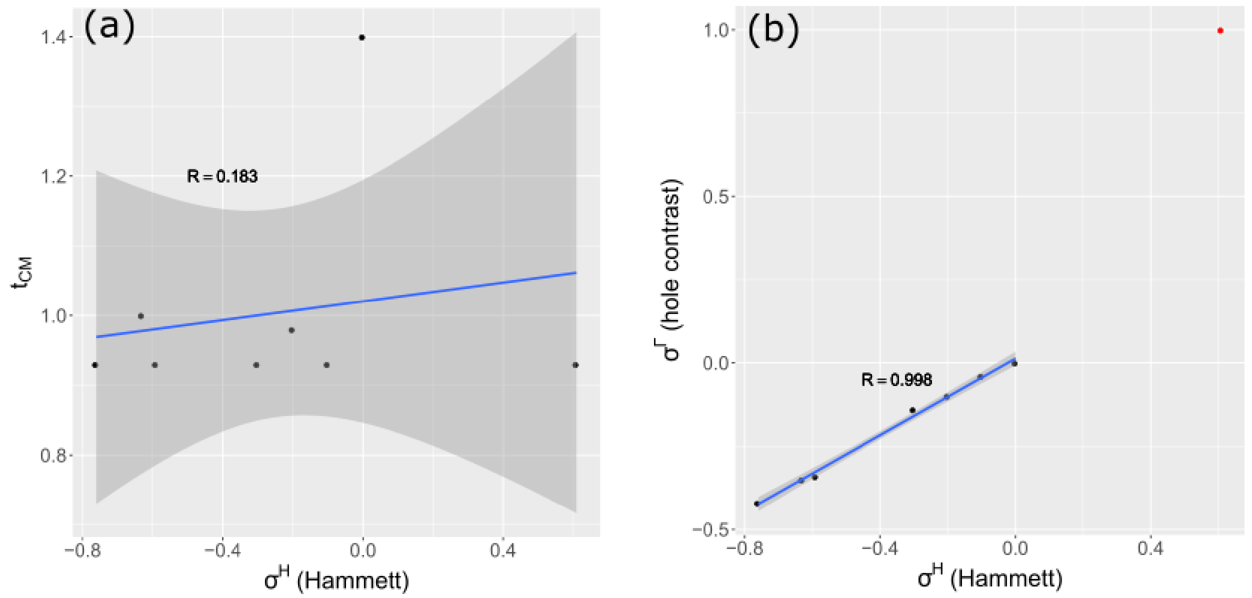

Figure S2: (a) The correlation plot between t_{CM} and σ^{H}. The R value of 0.183 shows that the charge migration time and Hammett value are not correlated. (b) The correlation plot between σ^{H} and σ^{Γ}. The electron-withdrawing group $-\mathrm{CF}_3$, which does not support CM, is shown as a red dot and not included in the fit. The R value of 0.998 means the contrast and Hammett σ values are highly correlated.

decrease in t_{CM} for the CM supporting molecules ($-\mathrm{CH}_3$,$-\mathrm{NH}_2$).

Real-time Time-Dependent Density Functional Theory

Real-time time-dependent density functional theory (RT-TDDFT) is a method used for propagating the electron density by integrating the time-dependent Kohn-Sham (TDKS) equations:

$$i\frac{\partial \psi_i(r,t)}{\partial t} = [-\frac{1}{2}\Delta^2 + v_{\mathrm{ks}}[\rho](r,t)]\psi_i(t) = [-\frac{1}{2}\Delta^2 + v_{\mathrm{ext}} + v_{\mathrm{H}} + v_{\mathrm{xc}}[\rho](r,t)]\psi_i(t) \quad (2)$$

where, $v_{\mathrm{ks}}(r,t)$: effective potential described by the time-dependent charge density ρ(r,t), $v_{\mathrm{ext}}(r,t)$: nuclear-electron; $v_{\mathrm{H}}(r,t)$: electron-electron mean field potential, and $v_{\mathrm{xc}}[\rho](r,t)$: exchange correlation potential. For $v_{\mathrm{xc}}[\rho](r,t)$ functional, we assume locality in time by using its adiabatic approximation.

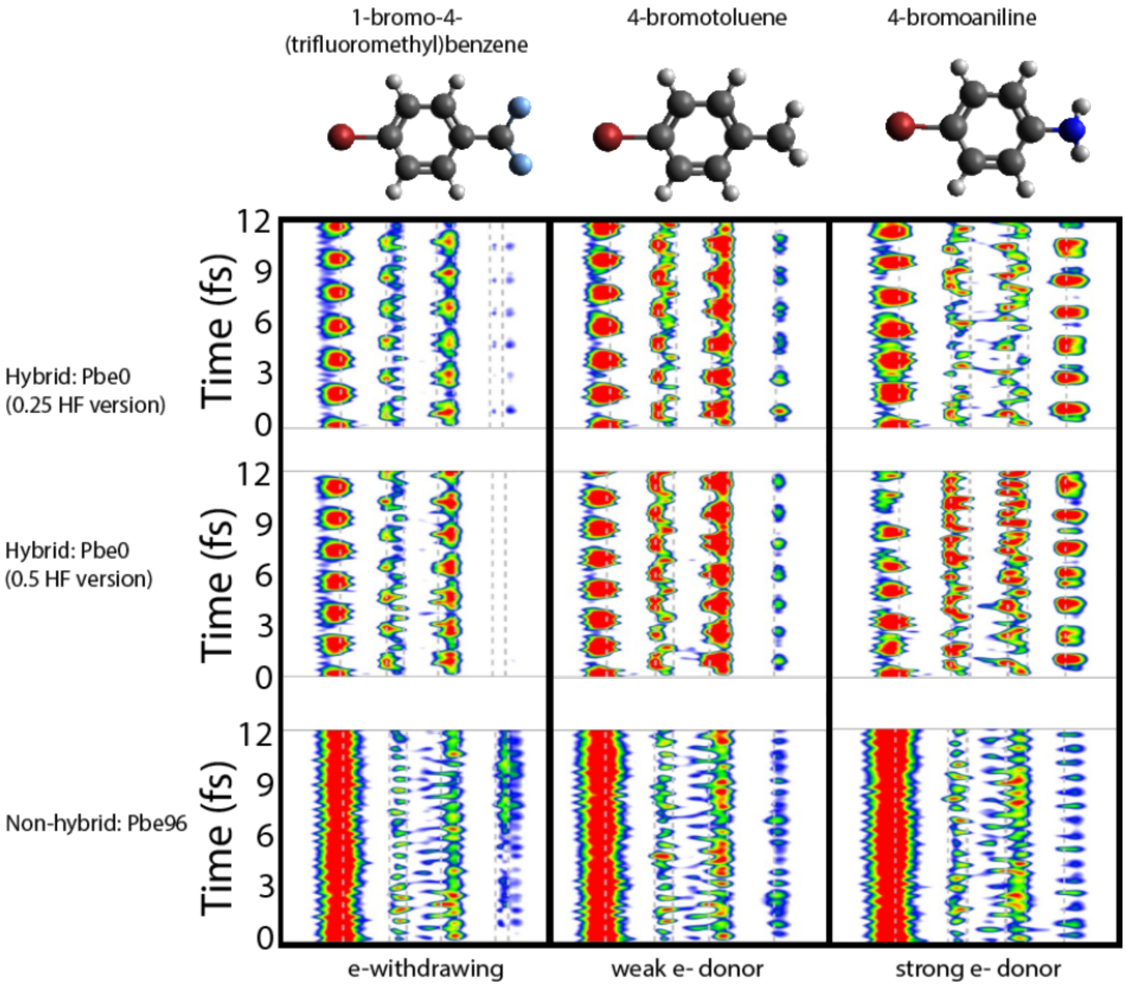

Figure S3: The effect of DFT exchange functional on CM dynamics in 1-Bromo-4-(trifluoromethyl)benzene, 4-bromotoluene and 4-bromoaniline. It shows the positive part of the time-dependent perpendicular-integrated hole densities using different (0.25HF, 0.50HF and no HF) Pbe0 exchange functionals. There is a good agreement between the results in 0.25 HF and 0.50 HF which means CM is insensitive to the amount of HF. The non-hybrid functional, Pbe96 do not exhibit CM.

In this work we use two different basis sets: (i) nonorthogonal atom-centered orbital (AO) Gaussians for constructing the operators such as the Fork matrix, and (ii) orthogonal canonical orbital (CO) basis for time propagation. For these basis sets conversions (i.e. AO↔CO), canonical orthogonalization of the Fock (F) and density (P) matrices is used:

$$
\begin{aligned}
F' &= X^{\dagger} F X \\
F &= Y F' Y^{\dagger} \\
P' &= Y^{\ddagger} P Y \\
P &= X P' X^{\ddagger} \\
X &= U s^{-\frac{1}{2}} \\
Y = X s &= U s^{\frac{1}{2}}
\end{aligned}
$$

CO: primed notation; AO: unprimed notation; X and Y: transform matrices U: eigenvector and s: diagonal eigenvalue matrix

Generally, the self-consistent field (SCF) approach is used to initially converge the system to the ground state, after which the single-particle density matrix is time propagated in the CO basis (prime notation). Our simulations use a density matrix computed via constrained DFT instead of the ground state. The equation of motion in the von Neumann model is given by:

$$
i \frac{\partial P'(t)}{\partial t} = [F'(t), P'(t)]
$$

$F'(t)$ and $P'(t)$ are the Fock and density matrices in the orthogonal canonical orbital basis. Using a second-order Magnus (exponential midpoint) propagator, we integrate equation of

motion:

$$P'(t+\Delta t) = e^{\Omega} P'(t) e^{-}\Omega$$
$$\Omega \equiv iF'(t+\frac{\Delta t}{2})\Delta t$$

By extrapolating the previous two times, propagating forward, and interpolating up until the propagated value is self-consistent, the time-advanced Fock matrix is calculated.

Geometries

All molecules are aligned such that the CM axis (X→R) lies along the $+x$-direction. All positions are in Angstroms.

```
1-Bromo-4-(trifluoromethyl)benzene
 Br                     0.00000000     0.00000000     0.00000000
 F                      6.73517265    -1.04289535     0.69663280
 F                      6.71546133    -0.08923709    -1.25778336
 F                      6.74367972     1.12481792     0.54645640
 C                      6.20310870     0.00000000     0.00000000
 C                      3.97959020    -1.20709898    -0.00813882
 C                      4.69291436     0.00290802     0.00651705
 C                      2.58241018    -1.20817007    -0.01107518
 C                      3.97834874     1.21177880    -0.00488601
 C                      2.58108986     1.21119415    -0.00764418
 C                      1.89015736     0.00099864    -0.00710977
 H                      4.50986367     2.16286722    -0.00679403
 H                      2.04610395    -2.15398734    -0.01550528
 H                      4.51302947    -2.15698813    -0.01245895
 H                      2.04363343     2.15624073    -0.00930622

1-Bromo-4-(trimethylsilyl)benzene
 Br                     0.00000000     0.00000000     0.00000000
 Si                     6.60924074     0.00000000     0.00000000
 C                      3.98333017     1.19283682     0.08001650
 C                      2.58587904     1.19700924     0.08000057
 C                      1.89040323    -0.00618819     0.00072863
 C                      2.57809361    -1.21417453    -0.07852225
```

C	3.97600976	-1.22122054	-0.07850356
C	4.69691477	-0.01735602	0.00119930
C	7.20029867	0.93376386	-1.51901313
C	7.20823482	0.87433358	1.55126593
C	7.29870516	-1.74399334	-0.03363628
H	4.50327780	2.14767441	0.14196751
H	2.05328685	2.14214721	0.14167617
H	2.03860679	-2.15543275	-0.14097458
H	4.48440123	-2.18128064	-0.14216011
H	6.84293790	0.45008370	-2.43298015
H	6.83340271	1.96439684	-1.50952216
H	8.29355637	0.96085943	-1.55113292
H	6.84655025	1.90636875	1.58161759
H	6.85144861	0.35833364	2.44760098
H	8.30172217	0.89530766	1.58096923
H	6.96583577	-2.31219788	0.84009547
H	6.97576226	-2.27472807	-0.93422128
H	8.39270164	-1.72272894	-0.02735015

4-Bromotoluene

Br	0.00000000	0.00000000	0.00000000
C	1.89050605	0.00383252	0.00096907
H	2.04205415	2.09302402	0.52984434
H	2.05151193	-2.08477904	-0.52775949
C	2.57966270	1.17762969	0.29811366
C	2.58465001	-1.16699747	-0.29542478
C	3.97654364	1.18019834	0.29876384
C	3.98117110	-1.16253217	-0.29429449
H	4.50447869	2.10220109	0.53216738
H	4.51697151	-2.08045488	-0.52666499
C	4.68788474	0.01060455	0.00268323
C	6.18825226	0.00000000	0.00000000
H	6.56136325	-0.71576996	0.73951987
H	6.56136496	-0.27769223	-0.99101059
H	6.59755726	0.98450952	0.24922552

4-Bromoanisole

Br	0.00000000	0.00000000	0.00000000
O	5.95410518	-1.06322769	0.01982005
C	6.89817351	0.00000000	0.00000000
C	4.63127063	-0.71733744	0.01195230
C	3.74591722	-1.79963187	0.01592899
C	2.36479967	-1.59194178	0.01144185
C	4.12183499	0.58262279	0.00288312
C	2.73857112	0.79542495	-0.00067108

C	1.86722615	-0.29206541	0.00374924
H	4.77097472	1.45179041	-0.00038460
H	4.13741516	-2.81426569	0.02262011
H	1.69400964	-2.44643216	0.01452438
H	2.35396404	1.81201774	-0.00632883
H	7.89920687	-0.44159879	0.01930029
H	6.81013085	0.58722028	-0.92003867
H	6.79818611	0.63510633	0.88638396

4-Bromophenol

Br	0.00000000	0.00000000	0.00000000
C	1.89051204	0.01326602	0.00000000
H	2.02820205	2.17053443	0.00000000
H	2.06386249	-2.14295340	0.00000000
C	2.57348311	1.23046575	0.00000000
C	2.59072085	-1.19280997	0.00000000
C	3.97021845	1.24259419	0.00000000
C	3.98610900	-1.17937960	0.00000000
H	4.49337981	2.19357640	0.00000000
H	4.54276345	-2.11239038	0.00000000
C	4.66412437	0.03544196	0.00000000
O	6.02675744	0.00000000	0.00000000
H	6.36306449	0.91117679	0.00000000

4-Bromoaniline

Br	2.19836981	0.09862975	-0.08045295
C	4.08718351	0.05617521	-0.03295101
H	4.19828404	-2.10104206	0.00230078
H	4.29514485	2.20618698	0.00220198
C	4.75223987	-1.16656524	-0.00427688
C	4.80653198	1.24769761	-0.00433329
C	6.14942104	-1.19303325	0.03678150
C	6.20349872	1.21141582	0.03672798
H	6.65719263	-2.15229343	0.09418370
H	6.75387026	2.14682646	0.09408394
C	6.88851813	-0.00686866	0.00478600
N	8.27705476	-0.03805396	0.17520829
H	8.71311392	-0.89063687	-0.16211133
H	8.75099649	0.79400935	-0.16214872

4-Bromo-N-methylaniline

Br	0.00000000	0.00000000	0.00000000
C	1.88922992	0.01960477	-0.02219884
C	2.57079090	1.23164238	0.00115842
C	3.97107700	1.24378823	-0.00617893

C	4.70737098	0.05431937	-0.06894542
C	2.59500487	-1.17765008	-0.04546769
C	3.99351659	-1.15423953	-0.05702053
H	2.02903725	2.17278681	0.03688298
H	2.07521098	-2.13139630	-0.04262232
H	4.52990151	-2.10033772	-0.04803492
H	4.46967390	2.20716127	0.03739088
N	6.10116911	0.00000000	0.00000000
C	6.89162848	1.19191222	-0.24357073
H	6.67543085	1.62171963	-1.22707444
H	7.95414480	0.92941232	-0.21888571
H	6.72574889	1.94466025	0.53384740
H	6.47519531	-0.82899113	-0.45536100

4-Bromo-N,N-dimethylaniline

Br	0.00000000	0.00000000	0.00000000
N	6.12719342	0.00000000	0.00000000
C	6.86167116	1.24115413	0.22628936
C	6.87999469	-1.24159682	0.14091870
C	2.58342490	-1.20084208	0.05227532
C	3.98595835	-1.19463500	0.05774458
C	4.73378258	-0.00256335	0.03263097
C	3.98671973	1.19034231	0.00437334
C	2.58454473	1.19923233	0.00242584
C	1.88846218	-0.00070945	0.02260844
H	4.48265089	2.15620560	-0.02745633
H	2.05448372	-2.14984087	0.06575571
H	4.47830117	-2.16198114	0.07942214
H	2.05706780	2.14845263	-0.02298064
H	6.70224236	1.93936140	-0.60272213
H	6.56139352	1.71547848	1.16724354
H	7.94214358	1.06893600	0.28528039
H	6.56480035	-1.98663449	-0.59756331
H	7.94950834	-1.08135896	-0.03623048
H	6.76736445	-1.65396705	1.14925902

References

(1) Hamer, K.; Mauger, F.; Folorunso, A.; Lopata, K.; Jones, R.; DiMauro, L.; Schafer, K.; Gaarde, M. Characterizing particle-like charge-migration dynamics with high-order harmonic sideband spectroscopy. *Phys. Rev. A* **2022**, *106*, 013103.
